# Mapping the cell-membrane proteome of the SKBR3/HER2+ cell line to the cancer hallmarks

**DOI:** 10.1371/journal.pone.0272384

**Published:** 2022-08-01

**Authors:** Iulia M. Lazar, Arba Karcini, Joshua R. S. Haueis

**Affiliations:** 1 Department of Biological Sciences, Virginia Tech, Blacksburg, VA, United States of America; 2 Academy of Integrated Science/Systems Biology, Virginia Tech, Blacksburg, VA, United States of America; 3 Fralin Life Sciences Institute, Virginia Tech, Blacksburg, VA, United States of America; 4 Carilion School of Medicine, Virginia Tech, Blacksburg, VA, United States of America; University of Salerno, ITALY

## Abstract

The hallmarks of biological processes that underlie the development of cancer have been long recognized, yet, existing therapeutic treatments cannot prevent cancer from continuing to be one of the leading causes of death worldwide. This work was aimed at exploring the extent to which the cell-membrane proteins are implicated in triggering cancer hallmark processes, and assessing the ability to pinpoint tumor-specific therapeutic targets through a combined membrane proteome/cancer hallmark perspective. By using GO annotations, a database of human proteins associated broadly with ten cancer hallmarks was created. Cell-membrane cellular subfractions of SKBR3/HER2+ breast cancer cells, used as a model system, were analyzed by high resolution mass spectrometry, and high-quality proteins (FDR<3%) identified by at least two unique peptides were mapped to the cancer hallmark database. Over 1,400 experimentally detected cell-membrane or cell-membrane associated proteins, representing ~18% of the human cell-membrane proteome, could be matched to the hallmark database. Representative membrane constituents such as receptors, CDs, adhesion and transport proteins were distributed over the entire genome and present in every hallmark category. Sustained proliferative signaling/cell cycle, adhesion/tissue invasion, and evasion of immune destruction emerged as prevalent hallmarks represented by the membrane proteins. Construction of protein-protein interaction networks uncovered a high level of connectivity between the hallmark members, with some receptor (EGFR, ERBB2, FGFR, MTOR, CSF1R), antigen (CD44), and adhesion (MUC1) proteins being implicated in most hallmark categories. An illustrative subset of 138 hallmark proteins that included 42 oncogenes, 24 tumor suppressors, 9 oncogene/tumor suppressor, and 45 approved drug targets was subjected to a more in-depth analysis. The existing drug targets were implicated mainly in signaling processes. Network centrality analysis revealed that nodes with high degree, rather than betweenness, represent a good resource for informing the selection of putative novel drug targets. Through heavy involvement in supporting cancer hallmark processes, we show that the functionally diverse and networked landscape of cancer cell-membrane proteins fosters unique opportunities for guiding the development of novel therapeutic interventions, including multi-agent, immuno-oncology and precision medicine applications.

## Introduction

The cancer hallmarks were first described by Hanahan and Weinberg [1, 2], and evolved to include six fundamental biological capabilities that sustain neoplastic growth (sustained proliferative signaling, insensitivity to anti-growth signals, evasion of apoptosis, limitless replicative potential, sustained angiogenesis, tissue invasion and metastasis), two emerging hallmarks of a more general nature (reprogramming of energy metabolism and evasion of immune destruction), and two enabling characteristics (genome instability and inflammation). Genetic changes drive the formation of a primary tumor, however, cell-autonomous mechanisms propelled by a diverse mutational landscape are not sufficient to explain the full range of aberrant behaviors. For example, tissue invasion and metastatic propensity have been described as being driven by a supportive tumor micro- or systemic macro-environment and epigenetic re-programming rather than metastasis-specific driver mutations [3–7]. Mutations, on the other hand, have been shown to heavily affect the epigenetic regulators [8]. Also, synergistic effects enabled by cell-extrinsic environmental stimuli (signaling, growth, cytokine or angiogenic factors, recruited stromal cells, etc.) and cell-intrinsic EMT gene regulators have been proposed to drive the metastatic processes via adaptation rather than selection [8–11]. As a result, in support of the metastatic process, additional hallmarks that include microenvironment modulation, plasticity, motility/invasion, and colonization have been proposed [12]. Moreover, the presence of a tumor microbiome has been implicated in tumor-supportive inflammation and tumor progression [13]. Altogether, genetic and epigenetic alterations work in tandem with a cooperative environment to reprogram gene expression, corrupt biological regulatory pathways, mediate adaptation in support of malignant neoplastic growth and metastasis, and ultimately drive the evolution of drug resistance [8, 14].

Non-cell-autonomous mechanisms that support cancer development and steer its evolution act through the interface between the cancer cells and their environment. This interface is defined by the cell-membrane that imbeds proteins with critical roles in intra-cellular signaling and inter-cellular communication, cell-cell, cell-pathogen and immune recognition events, cell-adhesion and motility, and exchange of solutes and various factors [15]. Cell-surface receptor tyrosine kinases (RTKs) trigger vital signaling cascades that control proliferation, differentiation, growth and metabolism. RTKs initiate the signaling process and regulate intracellular events by binding various ligands such as growth factors, peptides and hormones. Aberrant or mutated expression of such receptors is often the driver of uncontrolled proliferation. In contrast, cytokine receptors initiate signaling processes through association with other non-receptor protein kinases, while membrane proteases support secretory, cell-cell signaling, and degradation functions by cleaving the membrane-bound proteins [15]. Plasma membrane proteins also interact with lipids and carbohydrates to sustain various transport and endo-/exocytic processes to ensure in-and-out shuffling of solutes, nutrients, hormones, growth factors, cytokines, and other signaling and ECM molecules. Cell-junction and adhesion proteins, on the other hand, are critical not just to determining the 3D architecture of cell conglomerates, but also to inter-cellular or cell-ECM communication, as well as functionality within a tissue [15]. As a result, in the context of disease, the study of an extensive catalogue of cell-membrane proteins through their collective involvement in disease mechanisms, rather than specific functional roles, would be more meaningful to developing effective cures. Following this line of thought, the defining role of protein-protein interactions (PPIs) in orchestrating the cell-membrane protein-triggered events has been recognized, and both experimental and computational efforts have been geared toward achieving a comprehensive mapping of the cell-membrane interactome and using it for predicting novel therapeutic targets [16–19].

In this work, SKBR3/HER2+ cells were used as a model system to explore the cell-membrane proteome through the perspective of cancer hallmarks. Cell-membrane proteomic data generated by complementary methods were analyzed and queried for the presence of proteins that could be correlated with the hallmark processes. The results were assessed in the context of emerging interest in novel therapeutic paradigms that seek a systems approach for identifying precision oncology drug targets or drug target combinations with synergistic effects. PPI network centrality measures indicated the presence of potentially novel and valuable cancer drug targets.

## Materials and methods

### Cell-membrane protein fraction preparation and analysis

A detailed description of the experimental conditions that were used for generating the mass spectrometry (MS) raw files that were used in this study (cell culture, cell-membrane protein labeling/isolation, MS analysis), and of the procedure that was used for the annotation of membrane proteins, is provided in reference [20]. Briefly, SKBR3 breast cancer cells were acquired from ATCC (Manassas, VA), authenticated by STR (ATCC), grown either for 48 h in serum-free (McCoy 5A, Gibco, Carlsbad, CA) or 48 h serum-free/24 h serum-rich (10% FBS, Gemini Bioproducts, West Sacramento, CA) culture media at 37°C/5% CO_2_, and processed for generating cell-membrane enriched protein fractions. The SKBR3 cell-membrane proteins were isolated by either the biotinylation of amino (EZ-Link Sulfo-NHS-SS-Biotin, Thermo Fisher Scientific, Rockford, IL) or oxidized glycan (EZ-Link Alkoxamine-PEG4-Biotin, Thermo Fisher Scientific) groups followed by affinity NeutrAvidin (Thermo Fisher Scientific) pulldown, or by tryptic shaving of cell-surface proteins by using recombinant enzymes (TrypLE, Gibco). Three biological replicates of cells cultured in the presence or absence of serum, enriched in membrane proteins according to all three protocols, were generated. The tryptic proteolytic digests of the isolated proteins from all biological cell states and replicates were analyzed in triplicate by mass spectrometry using an EASY-nano liquid chromatography (LC) 1200 UHPLC/QE-Orbitrap-MS system (Thermo Fisher Scientific) and data-dependent higher energy collision (HCD)/MS2 data acquisition. The mass spectrometry raw files (total of 72) that were associated with the referenced work [20] and deposited in the ProteomeXchange Consortium PRIDE Archive (dataset identifiers PXD028976, PXD028977, PXD028978) were used in this study to map the detectable membrane proteins in the SKBR3 breast cancer cell line to the cancer hallmark processes.

### MS data processing

The LC-MS/MS raw files were analyzed by using the ProteomeDiscoverer 2.5 software package (Thermo Fisher Scientific), the Sequest HT search engine, and a reviewed, minimally redundant *Homo sapiens* database (DB) from UniProt with 20,433 entries (2019) [21]. The database searches were executed in a combined format for each set of raw files generated for each cell-membrane protein enrichment method. The MS searches were enabled for a parent peptide ion mass range of 400–5,000 Da, allowing for two missed tryptic cleavages, Met oxidation, Nt acetylation, and a biotinylation-induced modification of Lys residues (87.998 Da) for the case of amino labeled cell-membrane proteins. The quality of protein IDs that were mapped to the hallmark supportive DB was determined by: (a) the stringency of peptide identifications as defined by parent/fragment ion mass tolerances of 15 ppm and 0.02 Da, respectively; (b) the use of only rank 1 peptides and top scoring proteins; and (c) the use of a minimum number of two unique peptide matches to a protein sequence. All FDR targets for peptide spectrum matches, peptide and protein groups were set to either 0.03 (relaxed) or 0.01 (strict), and protein grouping was accomplished by using the strict parsimony principle. For data interpretation, three multiconsensus result files were generated for the database searches performed for each cell-membrane enrichment method (**S1 File**). Protein assignments to the cell-membrane or cell surface were made by using UniProt/GO annotations, the Human Protein Atlas cellular and organelle proteome database, and literature reports [21–23], as described in reference [20]. Protein abundances were assessed based on the summed peptide spectrum counts (SC), as log transformed values, i.e., log10 (SC).

### Bioinformatics data processing

The database defining the biological processes that support the cancer hallmarks was constructed by retrieving the relevant processes and pathways from the UniProt database by using the advanced search tool with the following options: (a) *Homo sapiens* organism, (b) only reviewed UniProt entries (i.e., Swiss-Prot entries), (c) GO annotation controlled vocabulary terms, and (d) any assertion method [21, 22]. The protein entries associated with these processes were extracted in the period July 2020-June 2021. Specific GO term definitions assigned to a particular cancer hallmark are provided in **Table 1**. The COSMIC (v94) Cancer Gene Census catalogue (CGC) [24], along with other hallmark proteins reported in the literature [1, 2, 25–29], were used for generating a better curated definition of hallmark proteins. Experimentally measured cell-membrane proteins were mapped to the cancer hallmark processes by aligning the list of detectable SKBR3 membrane proteins with the protein entries from the hallmark DB (**S2 File**). The circular data plot representing the detected SKBR3 cell-membrane proteins mapped to the cancer hallmarks and the corresponding genes within the 23 chromosomes in the human genome was created using the Galaxy platform [30] and the Circos data visualizing package [31]. Gene start and gene end positions were determined based on Ensemble gene annotations. Protein-protein interaction networks were created in STRING [32]. All interactions sources were enabled and the minimum required interaction scores were set to a confidence level of high or very high (i.e., 0.7/0.9). Network analysis was performed with the Cytoscape NetworkAnalyzer plugin. Network visualization and attribute circle layouts based on degree and betweenness centrality measures were created with Cytoscape 3.9.1 tools [33]. Cancer drug targets were extracted from DrugBank. This work did not involve the use of human subjects, and did not require IRB approval.

**Table 1:** Cancer hallmarks defined by GO biological processes and examples of cell-membrane proteins associated with the hallmarks.

Cancer hallmarks [1, 2]	Biological processes associated with the cancer hallmarks based on UniProt/GO annotations	# Protein IDs -Total/genome -Cell-membrane -Detected	Hallmark proteins associated with the cell membrane [1, 2, 25–29]	Cell membrane proteins present in the CGC with cancer promoting (↑), suppressing (↓), or dual role (↑↓).
Sustained proliferative signaling	• Cell communication (& regulation) • Signaling (& regulation) • Signaling receptor activity (& regulation)	6871 4058 847	RRAS, NRAS, HRAS, KRAS, EGFR, ERBB2, FGFR1/2, MAP2K1/2, MTOR, MET, IGF1R, PIK3CA, PDGFRA, GRB2, ***ABCC1***	NRAS ↑, HRAS ↑, RAC1 ↑, CALR ↑, ACVR1 ↑, DNM2 ↑, EPS15 ↑, ERBB3 ↑, ERBB4 ↑, ERBB2 ↑, EZR ↑, FGFR3 ↑, FGFR1 ↑, NDRG1 ↑, NOTCH1 ↑, NOTCH2 ↑, EGFR ↑, IL6ST ↑, KRAS ↑, PRKAR1A ↑, GNA11 ↑, GNAQ ↑, GNAS ↑, JAK1 ↑, MTOR ↑, MET ↑, FGFR2 ↑, FGFR4 ↑, MYD88 ↑, CTNNB1 ↑, PLCG1 ↑, PIK3CA ↑, PDGFRA ↑, RET ↑
Tissue invasion and metastasis	• Cell adhesion (& regulation) • Cell motility (& regulation) • Actin cytoskeleton organization (& regulation) • ECM organization (& regulation) • Secretion • EMT (& regulation)	4650 2646 742	CD44, EPCAM, POSTN, TNC, LGALS1, ABCC1, PIK3CA, ***ABCB1***	RHOA ↑↓, NRAS ↑, NRAS ↑, RAC1 ↑, CALR ↑, ACVR1 ↑, DDX3X ↑↓, ATP1A1 ↑, BMPR1A ↑, DNM2 ↑, ERBB3 ↑, ERBB2 ↑, EZR ↑, FAT1 ↑↓, FGFR1 ↑, FHIT ↓, NDRG1 ↓, NOTCH2 ↑, EGFR ↑, KRAS ↑, PRKAR1A ↓, MTOR ↑, MET ↑, FGFR4 ↑, MYD88 ↑, APC ↑, CTNNB1 ↑, BCORL1 ↑, PLCG1 ↑, PIK3CA ↑, PDGFRA ↑, RET ↑
Evasion of immune destruction	• Immune system process • Innate and adaptive immune response (& regulation) • Antigen processing and presentation (& regulation) • Autophagy (& regulation) • Cellular senescence • Cellular response to hypoxia (& regulation) • Chemotaxis (& regulation) • Chemokine mediated signaling pathway (& regulation) • Chemokine production (& regulation) • (Myeloid/B-cell/T-cell activation & regulation are included)	4055 2272 631	CD274, ***SIGLEC6***	NRAS ↓, RAC1 ↑, B2M ↓, EGFR ↑, JAK1 ↓, MET ↑, CTNNB1 ↑, RET ↑
Insensitivity to anti-growth signals (evading growth suppressors)	• Cell cycle, cell division, cell growth (& regulation) • Cell population proliferation (& regulation) • Protein catabolic process (& regulation) • Protein folding (& regulation) • Protein targeting (& regulation) • Signaling pathways & their regulation (PI3K, TOR, Wnt, signal transduction by p53 class mediator)	4925 1951 625	APC	RHOA ↑, ATP2B3 ↑, DDX3X ↑, ERBB4 ↑, FAT1 ↑, NDRG1 ↑, NOTCH2 ↑, GNAS ↑, APC ↑, CTNNB1 ↑, PDGFRA ↑
Genome instability	• Cellular response to DNA damage stimulus (& regulation) Disease variant	4030 1659 513	COL7A1, FEN1, MSH6	USP8 ↓, RHOA ↓, NRAS ↑, RAC1 ↓, CLTC ↓, FHIT ↓, LMNA ↓, ERCC4 ↑↓, MET ↑, ATRX ↓, APC ↓, CTNNB1 ↓
Evasion of apoptosis (resisting cell death)	• Cell death (& regulation) • Cell aging (& regulation) • Autophagy (& regulation) • (Includes apoptosis, senescence, necrosis)	2602 1139 374	ITGB, ITGB4, ***ABCB1***	RHOA ↑↓, NRAS ↑, NRAS ↑, RAC1 ↑, CALR ↑, ACVR1 ↑, DDX3X ↓, ATP1A1 ↑, ERBB3 ↑, ERBB4 ↑↓, ERBB2 ↑, EZR ↑, FAT1 ↑, FGFR3 ↑, NOTCH1 ↑, NOTCH2 ↑, EGFR ↑, KRAS ↑↓, PRKAR1A ↑↓, JAK1 ↓, MTOR ↑, MET ↑↓, FGFR2 ↑, MYD88 ↑, APC ↓, CTNNB1 ↑, PLCG1 ↑, PIK3CA ↑, PDGFRA ↓, RET ↑↓
Inflammation	• Inflammatory response (& regulation) • NFKB signaling (& regulation) • I-kappaB kinase/NF-kappaB signaling (& regulation) • Receptor signaling pathw. via STAT (& regulation) • MAPK cascade (& regulation) • TNF mediated signaling pathway (& regulation) • Macrophage activation (& regulation) • Cellular response to cytokine stimulus (& regulation)	2391 1323 345	COL1A1, ABCC1/2/3, CFTR, ITGB4, ABCC6, ***DDR1***	RHOA ↓, NRAS ↑, IL6ST ↑, KRAS ↑, MYD88 ↑, RET ↑
Deregulating cellular energetics (reprogramming of energy metabolism)	• Gluconeogenesis (& regulation) • Glycolytic process (& regulation) • Canonical glycolysis • Pyruvate oxidation • TCA cycle • Oxidative phosphorylation • Electron transport chain • ATP metabolic process (& regulation) • Carbohydrate metabolic process (& regulation) • Lipid metabolic process (& regulation) • One carbon metabolic process • Choline metabolic process • Cellular response to hypoxia (& regulation) • Peroxisome proliferator activated receptor signaling pathway (& regulation) • Energy homeostasis	2300 880 292	ATP1B1, GAPDH, IDH2, PFKM, ATP6V1B1, SLC2A1, ***VDAC1***	USP8 ↑, DDX3X ↑, ERBB2 ↑, NOTCH1 ↑, EGFR ↑, PICALM ↑, PAFAH1B2 ↑, KRAS ↑, SDHA ↑, MTOR ↑, CTNNB1 ↑
Sustained angiogenesis	• Angiogenesis (& regulation) • VEGF signaling (& regulation)	504 294 98	HSPG2, THBS1, FLT1, ***NRAS*, *HRAS*, *KRAS***, ***AGRN***, ***SDC1/4***, ***LGALS1***, ***ABCB1***	NRAS ↑, RAC1 ↑, CALR ↑, DNM2 ↑, NOTCH1 ↑, EGFR ↑, KRAS ↑, SDHA ↓, MTOR ↑, MET ↑, CTNNB1 ↑, PLCG1 ↑, PIK3CA ↑, PDGFRA ↑↓
Limitless replicative potential	• DNA replication (& regulation) • Chromosome organization (& regulation) • Signal transd. by p53 class mediator (& regulation) Telomere maintenance (& regulation)	1497 251 87	***RAP1A*, *ITGB1*, *TPP1***	NRAS ↑, FGFR1 ↑, NDRG1 ↓, NOTCH1 ↑, KRAS ↑, CTNNB1 ↑

Note: Proteins highlighted in bold/italic are part of other hallmark categories, according to GO annotations, than reported in the literature.

## Results

**Fig 1:**
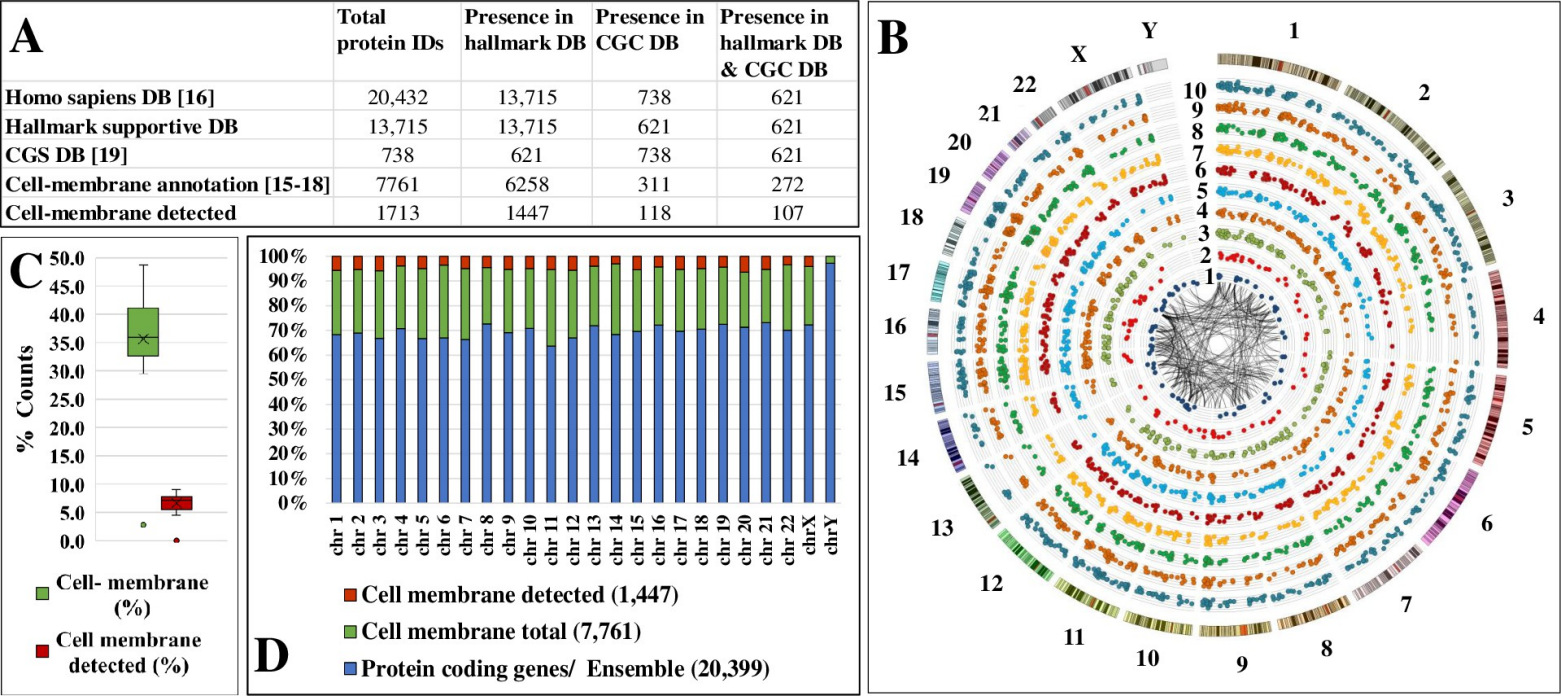
Cell-membrane proteins mapped to the cancer hallmarks. (**A**) Protein counts with hallmark association. **(B)** Circos plot of detected SKBR3 cell-membrane proteins mapped to their position in the human genome represented by 23 chromosomes, and categorized into 10 radially distributed cancer hallmarks. Hallmark categories, from inside-out (low-to-high counts): 1-DNA replication, chromosome organization, and telomeres; 2-Glycolysis, gluconeogenesis, and carbohydrate metabolism; 3-Angiogenesis; 4-Inflammatory response; 5-Cell death, apoptosis, senescence, aging; 6-Disease mutation; 7-Cell cycle division, growth, and proliferation; 8-Immune system processes; 9-Cell adhesion and motility; 10-Cell communication and signaling. The inner PPI network was constructed with proteins for which the STRING interaction score was ≥0.995. (**C**) Percent counts of cell-membrane proteins encoded on, and detected from, each chromosome. (**D**) Distribution of coded (total and cell-membrane) and detected proteins per chromosome.

Given the critical role of the plasma membrane in determining the fate of a cell, we hypothesized that many proteins that are integral to- or associated with the plasma membrane can be re-evaluated in the context of cancer hallmarks to support the detection and therapeutic treatment of cancerous cell states. To this end, an in-house database comprising ten broad categories of proteins that are either actively participating in-, or are just supportive or enablers of the cancer hallmarks was created by using the UniProt *Homo sapiens* DB and GO biological process and pathway annotations. **Table 1** provides the original hallmark categories, the biological processes and pathways associated with the specific hallmarks, and the number of proteins matched to each category. Overall, 6,258 cell-membrane/cell-surface proteins were associated with the hallmark DB (**Fig 1A** and **S2 File**). The number of experimentally detected cell-membrane proteins, and examples of cell-membrane hallmark proteins described in the literature [25–29] or reported in the CGC DB with cancer promoting, suppressing or dual role [24], are also provided. Decision for associating a particular biological process or pathway with a hallmark was made based on relevance and availability of adequate GO annotations, with the assumption that GO annotations are accurate.

To test our hypothesis, the dataset of SKBR3 proteins generated experimentally by the three complementary cell-membrane/cell surface enrichment processes [20] was queried for matches to the cancer hallmark DB. Mass spectrometric analysis yielded 3,263 cell-membrane proteins, with as many as 1,713 identified by high confidence two unique peptides, of which 1,447 could be matched to multiple hallmarks (**Table 1** and **S2 File**).

**Fig 2:**
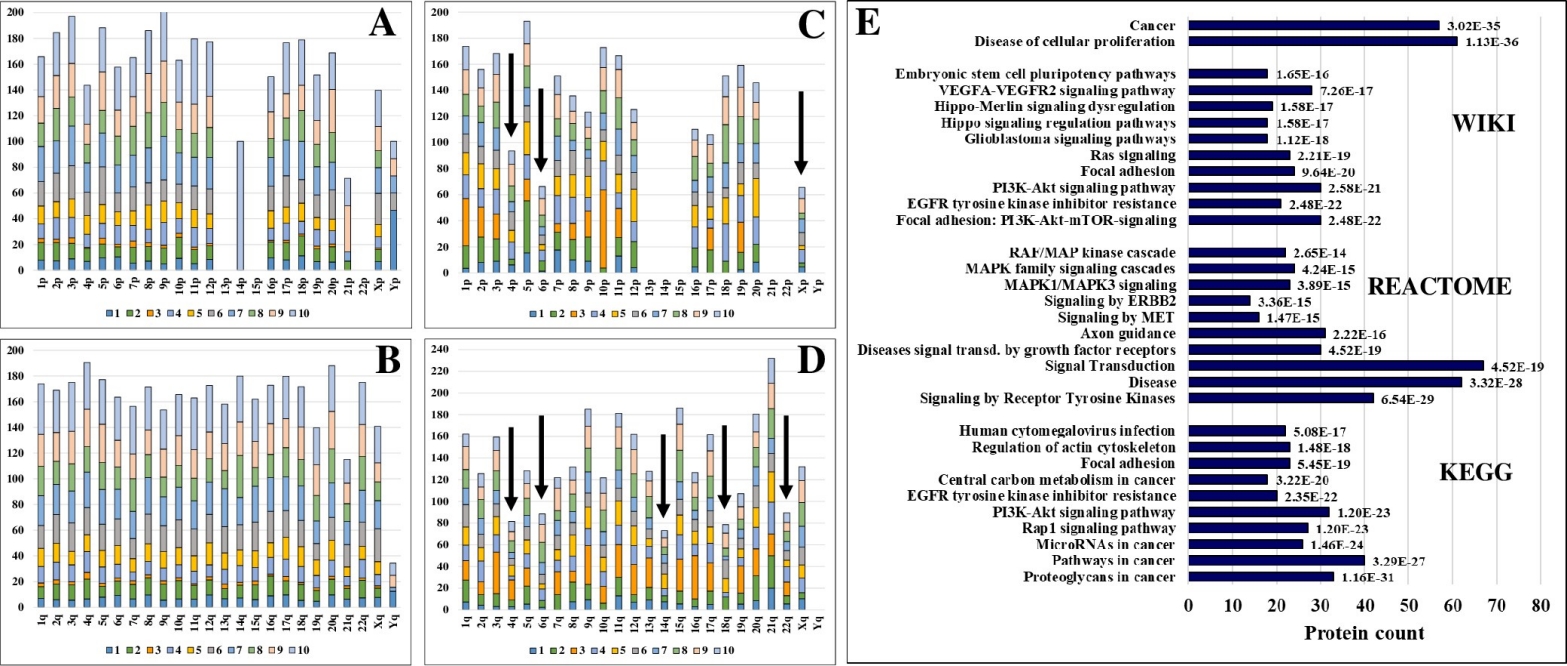
Stacked bar charts representing cancer-supportive human genes and proteins distributed per chromosome arms and per cancer hallmarks. The vertical ordering of hallmarks is following the same numerical trend as in Fig 1B (1-chart bottom, 10-chart top). (**A**) and (**B**) represent % cell-membrane protein-coding genes out of total coding genes per chromosome and p/q arms (the summed percentages exceed 100 because of genes with contributions to multiple hallmarks); (**C**) and (**D**) represent % cell-membrane proteins detected in SKBR3 out of total encoded; (**E**) Bar chart of top biological processes and pathways represented by the 138 subset of hallmark proteins (bar chart labels indicate FDRs).

A Circos plot with radially distributed hallmarks, in which each cell-membrane protein was mapped to its corresponding gene locus within the 23 human chromosomes, highlights the complex genetic landscape that can lead to cancer through various combinations of genes with altered functional products (**Fig 1B**). The cell-membrane proteins represent ~35–40% of the encoded proteins by each chromosome, with ~6–7% being detectable in SKBR3 when using the described experimental conditions (**Fig 1C** and **1D**). The hallmark-supportive proteins were encoded by the entire genome, excepting chromosome Y which did not have any matches because the SKBR3 cells originate from a female subject (**S2 File**). Protein abundance was represented by scatter plots depicting the log10(SC), with dot size representing a range spanning from 0 to 5. The non-coding centromeric areas and acrocentric chromosomal p-arms (13, 14, 15, 21, and 22) did not display any products (**Figs 1B, 2A** and **2B**), while some chromosomal coding regions appeared to be either under- (4p/4q, 6p/q, 14q, Xp, 18q, and 22q) or over-represented (21q) experimentally (**Fig 2C** and **2D**). The 17 proteins encoded by 21q were indicative of positive regulation of ROS metabolic and immune system processes, whereas angiogenic processes emerged more prominently across the entire spectrum as being supported by a range of receptors and adhesion proteins [e.g., THBS1, FGFR1/2, NOTCH1/2, TGFBR1, PDGFRA, ERBB2, CDC42, CAMs, integrins, and ephrins (e.g. EPHB4)]. The interpretation of such data calls, however, for prudence and further validation, as the SKBR3 cell line is highly aneuploid with numerous structural and numerical chromosomal aberrations (modal chromosome number 84), and the mechanistic impact of chromosomal alterations on protein expression and pathogenic outcomes still lacks a thorough understanding.

**Fig 3:**
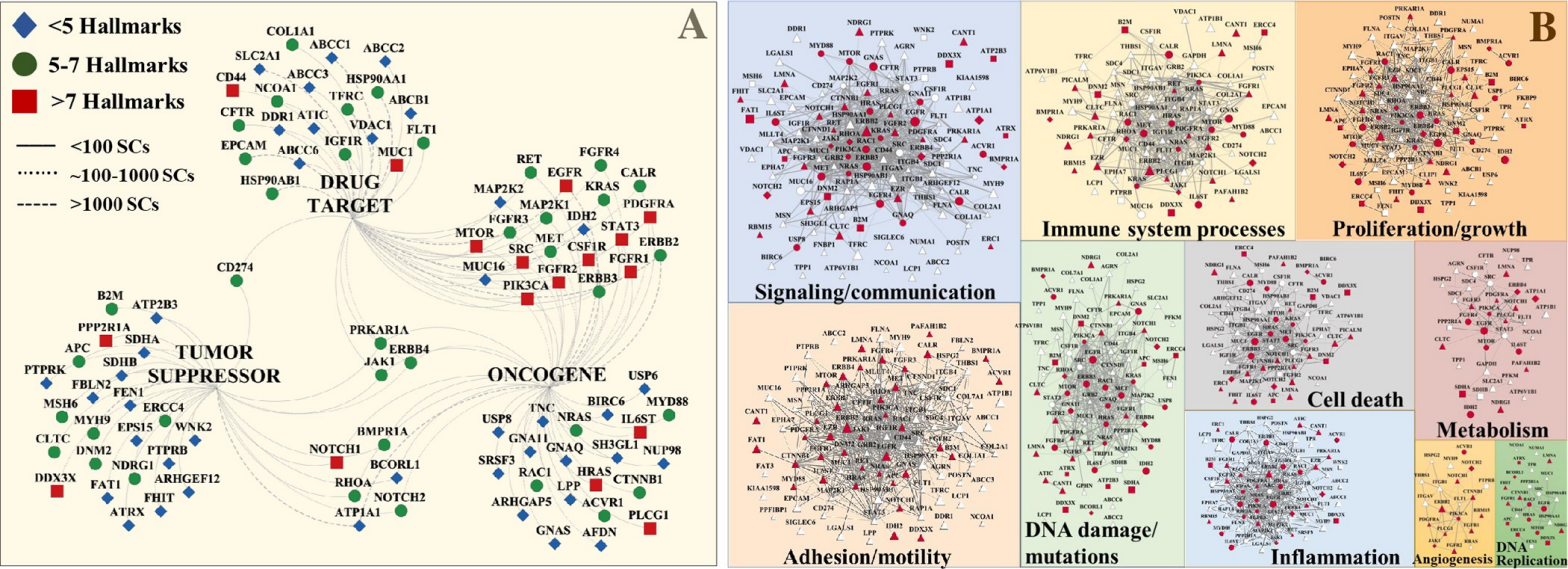
Drug targeting and PPI networks of 138 cell-membrane hallmark proteins. (**A**) Overlapping proteins between SKBR3 cell-membrane oncogenes, tumor suppressors, and drug targets. Top/left legend indicates the symbols of proteins matched to a particular number of hallmarks. Line width indicates the peptide spectral counts matched to each identified protein. (**B**) PPI networks constructed from 138 cell-membrane proteins matched to ten cancer hallmarks. CGC proteins are represented as oncogenes (◯), suppressors (◻), or oncogenes and suppressors (◊); Red icons indicate whether the protein was annotated as a cancer hallmark protein by CGC; Other hallmark proteins (△); Node size is proportional to the protein abundance; Edge thickness reflects the STRING interaction score (≥0.7).

To corroborate the relevance of these cell-membrane proteins to cancer, the COSMIC CGC list was queried against the hallmark database (**Table 1** and **S2 File**). The CGC comprises evidence-based, manually-curated information related to over 700 cancer-driving genes [24]. The majority of CGC proteins (621) could be mapped to the hallmark database, and encompassed 107 experimentally identified cell-membrane proteins of which 89 were tier 1 (i.e., with documented cancer-relevant activity and evidence of mutations that support oncogenic transformation) and 18 were tier 2 (i.e., with strong indications of cancer-related activity, but lack of sufficient evidence). The detected CGC cell-membrane subset also comprised 42 oncogenes, 24 tumor suppressors, and 9 proteins with overlapping oncogene/tumor suppressor roles. For further evaluation, the list of 107 CGC membrane proteins was supplemented with an additional 31 proteins with documented hallmark roles based on literature reports, for a total of 138 [1, 2, 25–29]. Not surprisingly, the top pathways that could be associated with this combined set of 138 representative proteins included cancer-enabling signaling (MAPK, PI3K/AKT, ERBB2, VEGF, HIPPO, EGFR tyrosine kinase inhibitor resistance, stem cell pluripotency) and migration supportive pathways (regulation of actin cytoskeleton, adhesion, ECM-receptor interaction, Rap1) (**Fig 2E**). Multiple small molecule or monoclonal antibody approved or investigational cancer drugs that target oncogenes or tumor suppressors from this list have been already described in the DrugBank database (**Fig 3A**).

**Fig 4:**
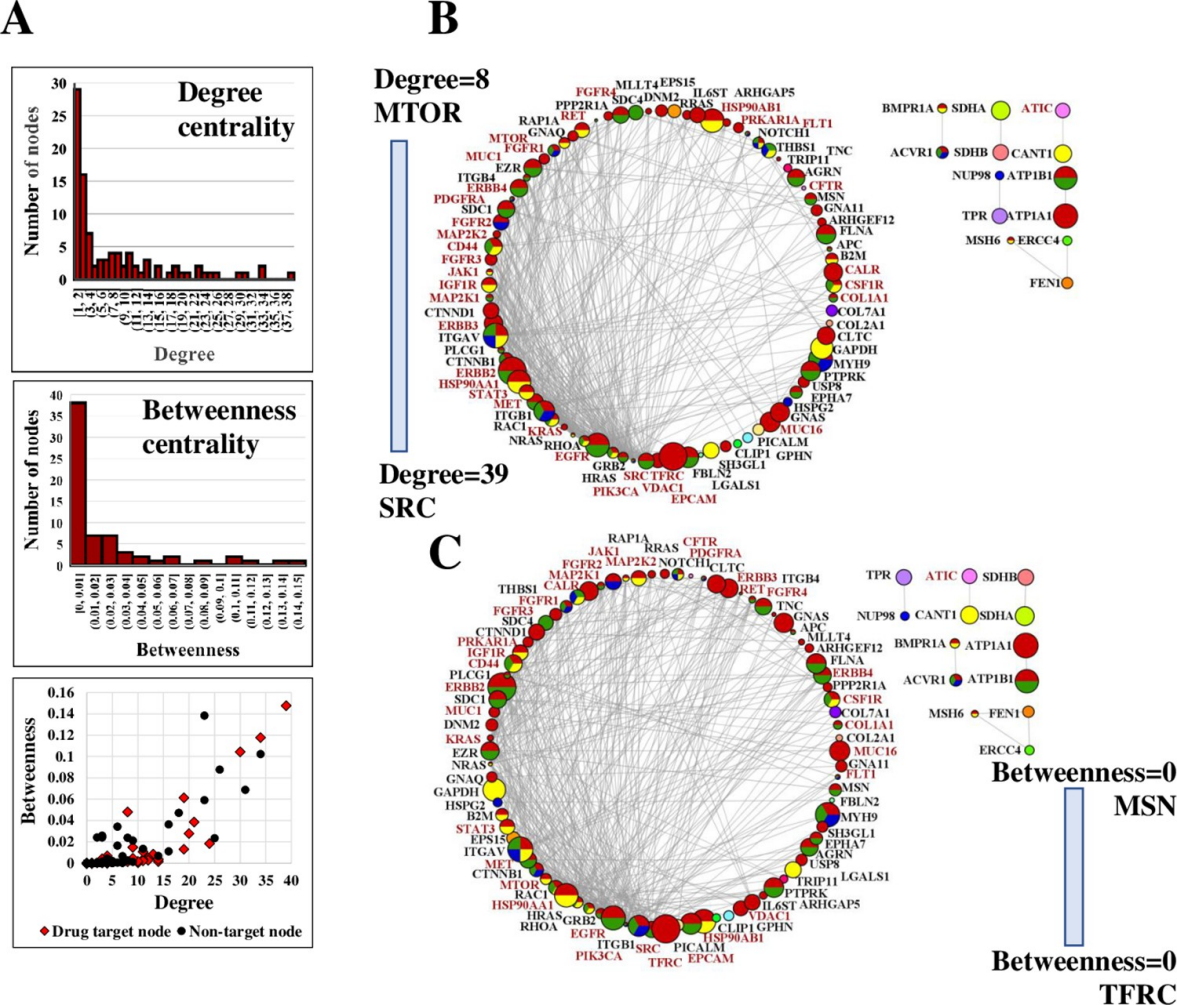
Network analysis of PPI networks created from 138 SKBR3 cell-membrane proteins (STRING interaction score ≥0.9). (**A**) Distribution and correlation of degree and betweenness centrality measures. (**B**) Degree-based circular layout PPI network (degree = 1–39). (**C**) Betweenness-based circular layout PPI network (betweenness = 5.51E(-5)-0.15). The circular distribution of attributes is presented in a clockwise fashion, from high (left) to low (right). Node size is proportional to the protein abundance and represented as LOG10(SC) over a range of 0.301–4.214. Node color coding: red-signaling, yellow-immune response, green-locomotion, blue-angiogenesis. Gene names that represent drug targets are shown in red. Network statistics: nodes 138 (only 97 nodes with degree ≥1 are shown), edges 397, avg # neighbors 9.262, network diameter 5, network radius 3, characteristic path length 2.406, clustering coefficient 0.466, network density 0.112.

PPI networks constructed based on the detected 138 subset cancer proteins (**Fig 3B**), or based on the whole set of 1,447 proteins (**S1 Fig**), underscored the intimate involvement of the SKBR3 cell-membrane proteome in each of the hallmarks and the progression of cancer. Network analysis of the 138 proteins (STRING interaction score>0.9) indicated node degree and betweenness centrality values ranging between 1–39 and 5.51E(-5)-0.15, respectively, both following the expected power-law distribution (**Fig 4A/**top two panels, and **S2 File**). Circular layout representations of degree and betweenness were created for the proteins that had interacting partners (i.e., 97 proteins with degree ≥ 1, out of 138), and revealed that the targets of the existing cancer drugs are proteins with rather high degree than high betweenness (**Fig 4B** and **4C**). With only 65 proteins displaying a betweenness value >0, a correlation between betweenness and degree was observable mainly for the higher range of the degree values (**Fig 4A**/bottom panel). Certain proteins, such as ERBB4, were characterized by a relatively high degree (= 10) but low betweenness (= 4.47E-4). Correlation between protein abundance and propensity for targeting was, however, not evident.

## Discussion

All major cell-membrane protein categories, i.e., with receptor/catalytic activity, adhesion/junction, transport, and CD classification, were represented in every cancer hallmark. The hallmarks of cell communication/signaling, adhesion/motility, immune response and cell cycle/growth accrued the largest number of protein hits, reflecting their considerable impact on cancer development and dissemination (**Table 1**). Some of the highest abundance membrane proteins were part of the very same categories, and included receptors, adhesion proteins, solute carriers, and transporters, as well as additional ephrins, nectins, integrins and essentially all detected CDs. A few specific examples, that also comprise hallmark proteins from the classic literature (highlighted in bold), include members with multiple roles in receptor mediated signaling (**ERBB2/CD340**, **EGFR**, TFRC/CD71, PTPRF, ITGAV/CD51, ITGB1, BCAM, PVR, SCARB1, SUSD2), adhesion (ITGAV, ITGB1/CD29, BCAM, **EPCAM/CD326**, PVR, SCARB1, MUC16, PVR, PTPRF, **EGFR**, **ATP1B1**, SCARB1), immune response (SUSD2, PVR, SCARB1, ITGAV, **EGFR**, TFRC/CD71, CD44), and transport (ABCC1, ATP1A1, **ATP1B1**).

The PPI networks from **Fig 3B** exposed highly interconnected protein clusters with several members playing roles in multiple hallmark categories (e.g., EGFR, ERBB2, FGFRs, CD44, SRC, STAT3). The emerging information can be used to identify novel regulatory or effector proteins, targets for knockout experiments, components of protein complexes, or to assign functionality to yet uncharacterized proteins. Through PPI networks, attention can be expanded to a vast set of membrane proteins that when targeted in a combinatorial or sequential fashion can impair cancer proliferation and dissemination not only through well-established mechanisms such as RAS/ERK or PI3K/AKT signaling, but also via processes implicated in angiogenesis, migration, cell death, metabolic reprogramming, inflammation, or remodeling of the tumor microenvironment. The ability to identify by MS aberrant protein isoforms or posttranslational modification patterns, e.g., glycosylation in the case of cancer [34], can further strengthen the efficacy of this approach. Pathways that confer intrinsic or acquired drug resistance and that often work via cross-talk or transactivation, or that lead to cancer stem cell mediated resistance, have been of particular interest to drug developers. Informed selection of drug targets, rather than based on empirical screening, will better suppress redundant or compensatory signaling pathways, prevent tumor-induced vascular remodeling, disable the ECM supportive environment, overturn immune evasion capabilities, and impair metastatic potential.

The SKBR3 cell-membrane landscape of oncogenes, tumor suppressors and existing drug targets revealed one such example of a group of key receptors (EGFR, ERBB, FGFR, IGFR, MET, MTOR, MAPK/RAS proteins), CD antigens (CD274, CD44), adhesion proteins (EPCAM, MUC1), and ABC transporters (ABCC1/2/3) that have been already explored for effective targeting of various cancers (**Fig 3A**). Such complex protein signatures and network architectures enable the targeting of broad panels of interconnected RTKs or CDs, and can uncover vulnerabilities that may trigger cascading failures from either within the established (**Fig 3B**) or the extended networks (**S1 Fig**). This, in turn, can enable combating widespread strategies used by cancer cells to resist the action of drugs, such as MET amplification or PI3K/AKT activation after targeted EGFR inhibition, PI3K/AKT compensation after mTOR inhibition, or MAPK/STAT3 cross-talk [14]. Targeting orthogonal pathways by using combinations of signal transduction or angiogenesis inhibitors (e.g., targeting Tyr kinase oncogenes), regulators of apoptosis (e.g., MUC1/16, a negative regulator of intrinsic apoptotic pathways), and inhibitors of drug efflux proteins (e.g., ABCC1 or multidrug resistance associated protein-MRP1), can be further explored to selectively attack or eliminate the cancer cells.

Network pharmacology, the topology of such networks, and the centrality of drug targets in these networks has been extensively researched to help identify and validate novel proteins amenable to therapeutic targeting [35–39]. In silico prediction of drug targets has been explored, for example, by using a multilayered interactome analysis coupled with a “guilt-by-association” approach [35]. While the known cancer drug targets in the circular diagrams from **Fig 4B** and **4C** were distributed along the entire range of degree and betweenness centrality values, the targets correlated better with high degree than high betweenness (note the drug target gene names shown in red). Most cell-membrane or cell-membrane associated drug targets (23 out of 35 targets from within the 97 proteins with degree ≥1) included proteins with a degree ranging from 39 (SRC) to 8 (MTOR), in the upper third of the degree distribution scale (**Fig 4B**). This highly interconnected set of 23 drug targets was involved mainly in positive regulation of kinase activity/intracellular signal transduction and transmembrane receptor protein tyrosine kinase signaling, the most represented KEGG pathways being EGFR Tyr kinase inhibitor resistance (16 proteins), ERBB (10 proteins), and PI3K-AKT signaling (18 proteins). In contrast, several targets fell in a region of the circular diagram where betweenness was minimal (**Fig 4C**). This result supports previous predictions that pinpointed that node degree in non-directed PPI networks is a better predictor of essentiality than betweenness, because there is no information flow through the nodes such as in the case of signal transduction networks [39]. Using centrality metrics in the context of a more limited but more fertile class of proteins, such as encompassed by the cell-membrane proteome, represents a promising alternative for identifying new drug targets. For example, several proteins for which drug antagonists have not yet been developed or approved, and that lined up in the circular diagram with degree>6, have been just recently suggested for consideration as molecular targets. Many of these included not just cell-membrane receptors involved in signaling, but also proteins with functionally diverse roles. Among these, EZR (degree = 9) has been proposed as a prognostic marker and target in acute myeloid leukemia [40] and SDC4 (degree = 7) for hepatocellular carcinoma [41]. Moreover, as aberrations in G-protein activating subunits have been shown to act as driver mutations implicated in multiple cancers [42], the use of siRNA to target and downregulate mutated GNAQ (degree = 8) in several cancers, in particular melanoma, has been also explored [43]. MUC1 (degree = 9), an epithelial membrane antigen with roles in signaling and cell-adhesion, has been just recently explored for the development of anti-MUC1 antibodies for targeted therapy of GI cancers and development of anti-cancer vaccines [44]. THBS1 (degree = 4), a cell adhesion glycoprotein that mediates cell-cell and cell-matrix interactions, has been suggested as a target for glioblastoma [45]. Silencing or knockdown of THBS1 in cell lines or mice resulted in impaired invasion and increased survivability, respectively, demonstrating its therapeutic potential. These novel trends underscore the power of orthogonal discovery efforts that can deliver valuable candidates for supporting the development of synergistic cancer therapeutic approaches that overcome the simplistic “one disease/one target/one drug” paradigm.

In contrast, the drug targets for which high confidence interacting partners did not emerge (degree = 0) were represented mostly by transport proteins (e.g., ABC transporters). These included, however, the multidrug resistance ABCB1 and the multidrug resistance associated ABCC1/ABCC6 transporters which were detected with a moderate number of spectral counts. As over-expression of a protein in a pathological condition does not necessarily make it a putative pharmacological target, further development of sensitive methods for the detection of low-abundance cell-membrane proteins and their PPI partners, as well as advancements in the understanding of drug action mechanism, uptake and efflux, will be essential to broadening the cell-membrane functional vista that is explored for the development of therapeutic drugs.

As a complementary undertaking, tumor-stroma interactions that support cancer progression and metastasis can be interrupted by disabling paracrine (FGF, Wnt, Hedgehog, TGFβ, NOTCH) and autocrine signaling sustained by cell-membrane receptors and proteases. Therapies aimed at modulating the tumor-immune cell dialogue, by using for example immune checkpoint inhibitors such as the anti-programmed cell death ligand 1 (PD-L1 or CD274) or anti-PD1 [46], with PD-L1 also detected among the SKBR3 membrane proteins, are expected to find a fertile ground in the rich backdrop of cell-membrane antigens. Such immunotherapies can be used alone or jointly with chemo- or targeted therapies. PD-L1 emerged without interacting partners in the network generated with the confidence score of 0.9, but had 4 interaction partners if the network was generated with a confidence score of 0.7, underscoring the need for accurately and thoroughly mapping the human interactome [17].

## Conclusions

In summary, mapping the SKBR3 cell-membrane proteome to cancer hallmark-supportive processes exposed a complex scenery of critical players that promote and sustain the development and metastatic propensity of these cells. The cell-membrane harbors an abundant supply of putative targets for the development of modern cancer therapies, and more extensive profiling of cell lines and tumors will help refine the list of viable candidates. Nevertheless, the molecular determinants of cancer are “unique” to each individual. By combining experimental findings with existing knowledge of fundamental mechanisms that drive the aberrant behavior of cancer cells, this work shows that the analysis of even one single cell line can be informative enough to uncover a large number of known oncogene and tumor suppressor drug targets, and to reveal through PPI network analysis that network centrality measures are useful indicators for the selection of new targets. Such results are particularly relevant to precision medicine approaches where pharmacological targets are being selected based on patient-specific, unique signatures of cancer supportive features. With the advance of high-throughput MS instrumentation, the ability to quickly generate tumor cell-membrane proteomes will create the necessary resource for exploring complex and aberrant protein expression profiles, studying additive or synergistic effects of drug combinations that target hallmark proteins, understanding the evolution of (multi)drug resistance, evaluating cancer risk and prognostic factors, and facilitating the selection of patient-tailored drug targets for achieving superior therapeutic outcomes in the various stages of cancer development.

## Supporting information

S1 File Proteome discoverer 2.5 result files comprising proteins matched by a minimum of two unique peptides. (XLSX)

S2 File Protein IDs associated with the cell-membrane and the cancer hallmark processes, detected cell-membrane proteins, and degree/betweenness centrality measurements. (XLSX)

S1 Fig Extended PPI networks mapped to the cancer hallmarks. (PDF)
