# Supplementary figures and images for: Mapping the cell-membrane proteome of the SKBR3/HER2+ cell line to the cancer hallmarks
Source: PLoS One. 2022 Aug 1;17(8):e0272384. doi: 10.1371/journal.pone.0272384 (PMC9342750; doi:10.1371/journal.pone.0272384)

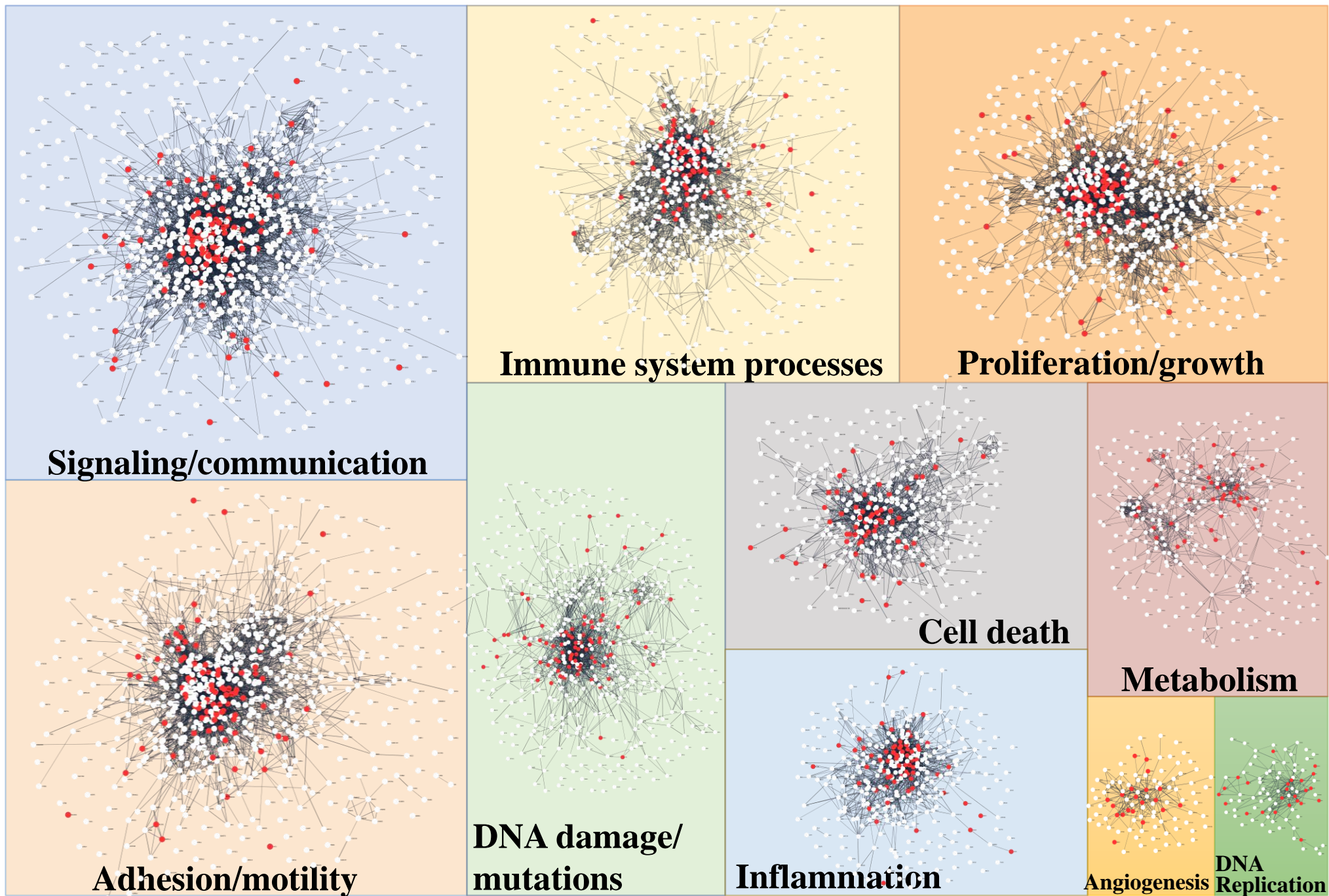

Supplement: S1 Fig — (PDF) [file pone.0272384.s003.pdf]
